# Appraisal of Hygiene Indicators and Farming Practices in the Production of Leafy Vegetables by Organic Small-Scale Farmers in uMbumbulu (Rural KwaZulu-Natal, South Africa)

**DOI:** 10.3390/ijerph10094323

**Published:** 2013-09-13

**Authors:** Fezile Mdluli, Joyce Thamaga-Chitja, Stefan Schmidt

**Affiliations:** 1 Discipline of Food Security, School of Agricultural, Environmental and Earth Sciences, University of KwaZulu-Natal, Private Bag X01, Pietermaritzburg 3209, South Africa; E-Mails: fezilemdluli@gmail.com (F.M.); chitjaj@ukzn.ac.za (J.T.-C.); 2 Discipline of Microbiology, School of Life Sciences, University of KwaZulu-Natal, Private Bag X01, Pietermaritzburg 3209, South Africa

**Keywords:** small-scale farmers, organic farming, food security, food safety, hygiene indicator organisms, coliforms, irrigation

## Abstract

During October, November and December 2011 (when highest sales of Agri-Hub fresh produce are observed), irrigation water, compost, lettuce and spinach sampled from four different farmer cooperatives supplying the local Agri-Hub in uMbumbulu (KwaZulu-Natal, South Africa) were analyzed monthly for the presence of total and fecal coliforms and *Escherichia coli* using the most probable number (MPN) technique. The pH values for all irrigation water samples analyzed were within the acceptable range of 6.5–8.5 for agricultural use. Fecal coliform levels were <1,000 MPN per 100 mL irrigation water and <1,000 MPN per g of compost. The vegetables produced by Agri-Hub small-scale farmers met the requirements for total coliforms of <200/g set by the South African Department of Health at the time of sampling. *E. coli* MPN values for irrigation water and vegetables were below the limit of detection. In addition, the farming practices of 73 farmers were assessed via a survey. The results revealed that more than 40% of farmers used microbiologically safe tap water for irrigation and that trained farmers have a significantly better understanding of the importance of production hygiene than untrained farmers. These results reiterate the importance of interventions that build capacity in the area of food safety and hygiene of small-scale farmers for market access of formal value chains.

## 1. Introduction

In South Africa, small-scale organic farmers are increasingly becoming important in food production. However, previously disadvantaged farmers have not participated in formal South African value chains and thus have limited knowledge of market specification and standards [1]. These small-scale organic farmers rarely supply larger markets, as a result of their inability to consistently produce large volumes of vegetables that meet various quality standards which include those of hygiene quality [1]. Organic farming (*i.e.*, farming without use of synthetic pesticides and chemical fertilizers; this farming may employ the use of livestock manure) in particular is said to have positive potential outputs for small-scale farmers world-wide, especially with consumer’s renewed interest in healthy foods such as fresh fruits and vegetables [2,3,4,5]. An additional advantage is the fact that the organic food sector might benefit from more loyal consumers [6] which would in turn enable small-scale farmers—which in South Africa are characteristically poorly skilled due to historical marginalization—to compete more successfully with conventional agriculture. However, organic farming is prone to microbiological contamination as seeds, irrigation water and manure are known as possible source of contamination in such farming systems [7,8]. Thus the low prevalence of *E. coli* in fresh produce analyzed recently in Germany was considered to be due to some degree to the fact that vegetables are mostly fertilized with synthetic fertilizers and not manure [9]. In addition, the absence of post-harvest chemical treatments of produce in organic farming may contribute to a higher microbial burden [10]. Though compost standards are not well defined, the importance of practicing correct composting techniques in order to decrease possible microbial contamination is acknowledged [11,12]. This is especially important in the production of leafy vegetables such as lettuce and spinach which are regarded as high risk produce often eaten without further processing [13].

Microbial contamination of river water sources has only recently been reported for South Africa [14,15,16]. In fact, river water in KwaZulu-Natal has been suspected to aid in the transfer of pathogens when overhead irrigation is used [15,17]. According to Berger *et al*. [3], drip irrigation may be a safer option when compared to sprinklers or spray irrigation as surface contamination and biomass build up is avoided. In addition, this will help to avoid internalization of potential pathogens present on the plant surface [18].

Pathogenic strains of *Escherichia coli* (*E. coli*) as well as *Salmonella* spp. and *Shigella* spp. have been associated with a large number of bacterial outbreaks [19,20,21,22]. *E. coli* outbreaks were reported in Japan in 1996 and the USA in 2006 due to contaminated radish sprouts and pre-packaged spinach, respectively [3]. More recently, Europe saw a severe outbreak of hemolytic uremic syndrome (HUS) caused by *E. coli* (STEC) [21,23]. Contaminated fenugreek seeds from Egypt were identified as the vehicles of transmission of STEC [23,24]. Consumers who are young, old, pregnant and immune-compromised are commonly identified under the acronym YOPI and are considered as being particularly vulnerable to such outbreaks as a result of their weakened immune systems. Organic farmer knowledge of microbiological standards is therefore important in not only minimizing such outbreaks but also meeting the stringent market standards [1]. Fecal coliforms are hygiene indicator organisms commonly used to determine the hygiene quality of water and produce as they are regarded as a reliable means of determining fecal contamination and the possible presence of enteric bacterial pathogens [25,26]. Among these so-called fecal coliform bacteria, highly pathogenic toxin forming strains of *E. coli* such as STEC (**S**higa **t**oxin producing *E. coli*) are a major concern [27]. Farmers need to be well informed of practices that may increase the risk of microbiological contamination of water and produce. The building of capacity in areas such as water safety, correct composting techniques and good personal hygiene all need to be addressed [1,28]. This study aimed to assess selected aspects of production hygiene and training of small-scale rural farmers involved in the organic production of vegetables.

## 2. Experimental Section

### 2.1. Site Location, Farmer Survey and Data Collection

The 4 small-scale farmer cooperatives Jabulani, Nungwane, Senzakahle, and Siyazenzela assessed in this study are all situated in uMbumbulu (KwaZulu-Natal, South Africa) within a 2 km vicinity of the Agri-Hub (29°59'27.96''S, 30°42'28.8''E). The survey interviewed 73 farmers living within the uMbumbulu vicinity. Some of these farmers were working with the uMbumbulu Agri-Hub, which focuses on providing training on all aspects of the organic farming value chain and also purchases farmers produce. The Agri-Hub then supplies vegetables under the “organically produced” and not “certified organic” label as the organization’s produce is yet to be formally certified as organic. This study worked with 33 farmers supplying the Agri-Hub and belonging to the Jabulani (12) Nungwane (3), Senzakahle (10) and Siyazenzela (8) cooperatives. The rest of the numbers (40) were made up of untrained farmers yet to supply the Agri-Hub. The questionnaires (available as supplementary online material) were prepared in IsiZulu and English which provided insight into farmers’ attitudes, behaviors, and general hygiene practices when farming. The questionnaire data were collected through face to face interviews.

### 2.2. Sample Collection

Water samples from four different locations (Jabulani, Nungwane, Senzakahle and Siyazenzela) were obtained monthly using sterile 1 L Schott bottles from areas of fast flow (for river water) at a depth half that of the total in order to avoid debris and collecting exclusively surface water. In the case of tap water, 1 mL of Na_2_S_2_O_3_ solution containing 18 mg of the pentahydrate was added to the sampling flask prior to autoclaving in order to neutralize the incoming free chlorine atoms usually found in the tap water. About 20 g of spinach, lettuce and compost samples were collected aseptically monthly at the four different study sites and placed into sterile Erlenmeyer flasks. Leaf samples were collected by removing not less than 20 g of produce material combined from at least three different plants. To avoid soil-based contamination, material closest to the soil surface was avoided. This sampling procedure mimicked the practice employed by farmers whereby the oldest outermost leaves exposed to soil are not harvested for consumption and for supplying the Agri-Hub. Compost from the top part of the compost heap was taken for analysis as farmers usually use this material for fertilization as stated by key informers. All samples were stored and transported on ice and analyzed in the laboratory within 2 h. Temperature and pH of water samples were measured on-site using a calibrated pH/°C meter (Hanna Instruments, HI8314, Padova, Italy). Chemical oxygen demand was determined in the laboratory using the Merck NOVA 60 system (Darmstadt, Germany) and a Merck COD test kit (25–1,500 mg/L, Merck) according to the manufacturer’s instructions.

### 2.3. Microbiological Analysis

Total and fecal coliforms as well as *E. coli* in water and produce samples were enumerated by using the MPN (most probable number) method MFHPB-19 according to Health Canada [29]. For water samples, each of five tubes of 10 mL double strength LST (Lauryl sulfate tryptose broth (Merck)), were initially inoculated with 10 mL undiluted irrigation water samples. The irrigation water samples were then diluted tenfold by aseptically pipetting 1 mL of the water sample into 9 mL of sterile 0.1% buffered peptone water (Merck) followed by subsequent decimal dilution (up to 10^−4^) using the same diluent. Produce and compost samples were prepared for analyses by adding 90 mL of 0.1% buffered peptone water solution to 10 g (fresh weight) of leaf/compost material in a sterile Erlenmeyer flask followed by a 10 min treatment on an orbital shaker (MRC) at 200 rpm and at ambient temperature prior to decimal dilution (up to 10^−8^) using the same diluent. Briefly, confirmation for total coliforms was done by inoculating Brilliant-green lactose bile broth (BGLB, Merck) using one loopful from gas-positive LST tubes. Fecal coliforms were quantified by inoculating gas-positive LST tubes into *E. coli* (EC) broth (Merck) followed by incubation at 44.5 °C. *E. coli* was confirmed using gas positive EC broth tubes obtained to inoculate Levine-eosin methylene blue (L-EMB) agar (Conda) and performing the prescribed biochemical confirmation tests—GIMViC (*i.e.*, gas production at 45 °C (G), indole formation from tryptophan (I), Methyl-Red (M), Voges-Proskauer (Vi) and Simmon’s citrate assimilation (C)). Results are expressed as MPN per 100 mL of irrigation water or g of compost or g of produce sample with a 95% confidence interval established as reported [30]. *E. coli* ATCC 8739, *Pseudomonas aeruginosa* ATCC 9027 and *Salmonella* Typhimurium ATCC 14028 were used as controls.

### 2.4. Survey Statistical Analysis

Questionnaire data were coded, captured and analyzed using the IBM SPSS 21 statistical package. Descriptive statistics such as the Chi-Square test evaluated the significance of relationships between practices and farmer training.

## 3. Results and Discussion

### 3.1. Farmer Responses on Vegetables Produced

**Figure 1:**
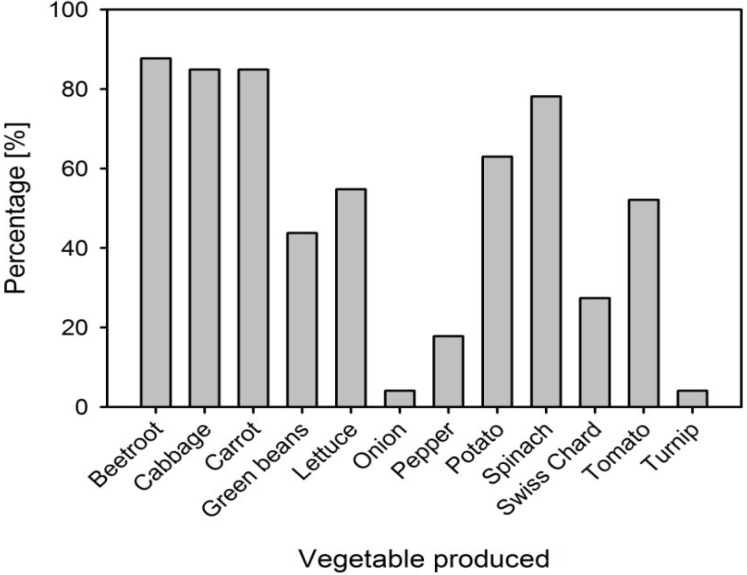
Most produced vegetables by farmers who participated in the survey (multiple vegetables per respondent, n = 73).

The questionnaire respondents produced an array of vegetables. These vegetables included beetroot, cabbage, carrot, green beans, lettuce, onion, pepper, potato, spinach, Swiss chard, tomato and turnip (Figure 1). All 73 respondents consumed the vegetables personally, with 33 (45%) of the farmers also selling surpluses to the community or the uMbumbulu Agri-Hub. A total of 87.7% of all farmers who participated in the study farmed beetroot and 84.9% produced cabbage and carrot. Onion and turnip were the least farmed vegetables as only 4.1% of the respondents produced them (Figure 1).

The choice of vegetables produced was largely motivated by personal preference, nutrition, health, customer demand, and affordability. Respondents cited that the affordability of seedlings and their understanding of the production system of vegetables resulted in the preference of certain vegetables over others. In this case, these farmers were well versed in using organic farming principles in their chosen vegetables. A similar study conducted in Embo, uMbumbulu, concluded that a farmer’s decision to produce organic vegetables was also influenced by perceptions that organic produce was more nutritious and safe [31].

Discussions with the key informants revealed that the primary objective for the farming of these vegetables was to improve the food and nutritional security of their families. This statement was confirmed by respondents who all admitted to consume the vegetables produced at a household level. Selling vegetable surplus for economic gain was encouraged only once the primary objective was fulfilled. The United Nations (UN) shares similar views, as it associates the implementation of small-scale organic farming with improved levels of household food security and the improvement of the quality of life [4,5]. There was a noted absence of traditional vegetables such as *amadumbe* (taro) and *ubhatata* (sweet potato) in the gardens. Farmers supplying the Agri-Hub attributed the lack of traditional vegetables like sweet potato to the fact that their market did not prefer these types of vegetables and subsistence farmers reported that the absence of traditional vegetables was a result of attack by *amaThendele* (wild birds) and *iMpunzi* (buck). The farmers produce vegetables that are demanded by the South African market, this is important in making their vegetables attractive to procurement specialists and organic produce supermarkets [1].

### 3.2. Irrigation Water Sources Identified in Farmer Survey

Questionnaire responses identified seven irrigation water sources (Table 1). The main irrigation water source supplying 42.5% of respondents was municipal supplied and treated tap water followed by river water (19.2%), dam water (16.4%) and spring water (9.6%). Borehole, wetland and tank water was the least used, with a total of 12.3% of the respondents using these sources; they were therefore not tested in this study. All farmers used watering cans to irrigate their produce. A total of 72.6% of respondents acknowledged water as a source of contamination in the farming environment. The water sources, frequency of use and percentage of the respondents utilizing them are tabulated in Table 1.

**Table 1:** Irrigation water sources in descending order as identified by the respondents.

Irrigation source	Frequency ∑ = 73	Percentage %
Tap water	31	42.5
IsiJodi River	14	19.2
Nongwane Dam water	12	16.4
Spring water	7	9.6
Borehole	4	5.5
Wetland	3	4.1
Tank water	2	2.7

Most respondents used tap water as a source of irrigation, the reason for this is that all the areas under study had access to municipal supplied running water. Some farmers preferred the use of natural water sources as they came at no financial cost, however, the natural water sources did not undergo any treatment prior to irrigation use. Discussions with the key informants revealed that the Agri-Hub thoroughly rinsed vegetables prior to sale using local tap water as a form of mitigation against possible unsafe material and due to concern and uncertainty on whether natural water sources met water quality standards.

### 3.3. The Importance of Farmer Training

The Agri-Hub provided training to all interested farmers in an attempt to improve quality of their produce. Only farmers who had received this training were allowed to supply the Agri-Hub. Farmers received training on farming practices that included composting, hygienic practices and soil management. Table 2 reveals the practices and knowledge of farmers belonging to the Agri-Hub (out of 33) and those yet to join (out of 40).

The difference in numbers between trained and untrained caused a distinct variation in the farming practices of the two groups. According to key informants, farmers who have attended at least two or more workshops were eligible to supply the uMbumbulu Agri-Hub and were thus registered in the farmer database. Trained farmers (*i.e.*, farmers that are part of the Agri-Hub) appeared to be more knowledgeable on practices that could introduce contamination into their gardens. In addition, Agri-Hub members practiced good hygiene and composted manure before use. Farmers not supplying the Agri-Hub were unclear on practices introducing microbiological contamination into gardens and avoided making compost heaps because of its physically demanding nature. Instead, farmers that were not a part of the Agri-Hub mainly used dried or wet manure from different animals in their farming practices. The Chi-square test indicated that trained Agri-Hub members were significantly more likely to carry out good farming practices and had better farming knowledge than those who were non-members of the Agri-Hub.

**Table 2:** Relationships existing between practices and training of small-scale organic farmers.

	Agri-Hub members (% n = 33)	Non-Agri-Hub members (% n = 40)	*p*-value	
*Hygienic practices prior to entering the garden*				
Individuals washing hands and boots	91	53	<0.001 ***	
Individuals washing of farming equipment	91	55	<0.001 ***	
*Individuals who acknowledge the following sources of contamination*				
Contaminated water	82	65	0.026 **	
Incorrect composting techniques	94	53	<0.001 ***	
Poor personal hygiene	91	23	<0.001 ***	
Contaminated soils	82	50	0.001 ***	
Contaminated equipment	85	50	0.002 ***	
*Type of treatment manure subjected to*				
Drying of manure (*umquba*)	55	48	0.305	
Composting	76	33	<0.001 ***	
Direct use of wet/fresh manure (no treatment)	9	38	0.009 ***	

** and *** show significant relationships at 5% and 1% significance levels respectively.

### 3.4. Physico-Chemical Characteristics of Irrigation Water Sources Tested

Analysis of water samples over three months (data summarized in Table 3) revealed that the highest water temperature observed was for spring water with 23.9 °C in October 2011 and the lowest for tap water with 18.0 °C in November 2011. Conversely, the lowest pH was detected in spring water (6.50, October 2011) and the highest pH of 7.79 in tap water (November 2011). The detected chemical oxygen demand (COD) values oscillated within a range of 39 mg/L for October 2011 (spring water) and 14 mg/L (dam water, October 2011 and tap water, November 2011).

**Table 3:** Physico-chemical characteristics of irrigation water sources tested in the months of October, November and December 2011.

Month		Irrigation Water Source			
		Nungwane Dam water	Spring water source	IsiJodi River water	Tap water
October 2011	Water temp.	23.1 °C	23.9 °C	18.4 °C	19.0 °C
	Water pH	7.72	6.50	7.64	7.34
	Water COD	14 mg/L	39 mg/L	25 mg/L	17 mg/L
November 2011	Water temp.	23.8 °C	22.0 °C	19.3 °C	18.0 °C
	Water pH	7.15	6.51	7.13	7.79
	Water COD	17 mg/L	36 mg/L	36 mg/L	14 mg/L
December 2011	Water temp.	23.5 °C	23.0 °C	19.8 °C	18.8 °C
	Water pH	7.52	6.54	7.45	7.66
	Water COD	18 mg/L	36 mg/L	17 mg/L	17 mg/L

The pH values observed for the water sources were all within the acceptable range of 6.5–8.5 stipulated for agricultural use by the South African Department of Water Affairs [32]. The elevated temperatures of the Nungwane Dam and the spring water when compared to river and tap water might be due to the fact that these water sources are stagnant. The chemical oxygen demand (COD), an indicator of organic pollutants in water, showed values of between 14 mg/L and 39 mg/L (Table 3), indicating that the chemical burden of the water was at the lower end of the range reported recently for two local rivers in KwaZulu-Natal [15,16].

### 3.5. Hygienic Quality of Irrigation Water

Following the physico-chemical analysis, the microbial burden was established for the irrigation water by targeting selected bacterial hygiene indicators. The MPN for total coliforms in the dam, spring, and river water ranged from 7.90 to 110.00 per 100 mL while fecal coliform MPN values ranged from 2.00 to 27.00 per 100 mL (Table 4). Tap water samples showed neither detectable total or fecal coliforms nor *E. coli* as was expected since tap water has to meet the strict South African drinking water requirements (no detectable fecal coliforms per 100 mL). The fecal coliform levels observed for the Nungwane dam, spring water and IsiJodi River were meeting the South African and WHO standards for the safe use of water for irrigation of <1,000 fecal coliforms per 100 mL [25,32].

**Table 4:** Most probable number (MPN) per 100 mL of irrigation water for total and fecal coliforms as well as *E. coli* in the irrigation water sources for the months of October, November and December 2011.

Source of irrigation water	October 2011		November 2011		December 2011	
	MPN/100 mL	95% confidence interval lower/upper limit *	MPN/100 mL	95% confidence interval lower/upper limit	MPN/100 mL	95% confidence interval lower/upper limit
*Total coliforms*						
Nungwane Dam	7.90	2.4/25	7.90	2.4/25	7.90	2.4/25
Spring water	110.00	39/300	79.00	25/247	110.00	39/300
IsiJodi River	110.00	39/300	25.00	11/62	33.00	11/99
Tap water source	n.d	-	n.d	-	n.d	-
*Fecal coliforms*						
Nungwane Dam	2.00	0.28/14	4.50	1.1/18	4.50	1.1/18
Spring water	14.00	5.5/34	4.50	1.1/18	4.50	1.1/18
IsiJodi River	27.00	11/64	4.50	1.1/18	7.90	2.4/25
Tap water source	n.d	-	n.d	-	n.d	-
*E. coli*						
Nungwane Dam	n.d	-	n.d	-	n.d	-
Spring water	n.d	-	n.d	-	n.d	-
IsiJodi River	n.d	-	n.d	-	n.d	
Tap water source	n.d	-	n.d	-	n.d	-

* 95% confidence limits calculated according to Garthright and Blodgett [30] n.d—not detected (limit of detection is 2/100 mL).

As the levels detected were well below the limit set by these regulatory bodies, it appears that all the analyzed irrigation water sources were microbiologically suitable for the irrigation of vegetables at the time of sampling.

### 3.6. Hygienic Quality of Compost

Only 71% of the 73 respondents were reported to compost animal manure and other organic matter. All composts prepared contained livestock manure, constituting as much as 1/3 of the compost heap. Respondents used manure from cattle, chicken and sheep, with some respondents using a combination of manure from these animals. A total of fifty two (71%) of the total respondents were aware of the fact that compost could be a source of microbiological contamination (see Table 2). Laboratory analysis was therefore undertaken to establish the number of total and fecal coliforms as well as *E. coli* present in compost. The MPN for total coliforms in the compost from the four different locations ranged from 22.10 to 1,405.60/g and the highest MPN for fecal coliforms was established as 313.90/g.

The highest *E .coli* levels were recorded for Nungwane in October (27.80/g). It is evident from Table 5 that the compost from Nungwane and Senzakahle cooperatives had the highest total coliform values with about 1,405.60/g in the month of October 2011. The Siyazenzela cooperative had the lowest levels of total coliforms which remained at about 22.12/g for all three months. The month of October 2011 had the highest abundance for total and fecal coliforms and *E. coli* in compost samples, all of which decreased from October to December 2011.

**Table 5:** MPN per g of compost for total and fecal coliforms as well as *E. coli* in the compost of the different farmer groups for the months of October, November and December 2011.

Source of Compost	October 2011		November 2011		December 2011	
	MPN/g	95% confidence interval lower/upper limit *	MPN/g	95% confidence interval lower/upper limit	MPN/g	95% confidence interval lower/upper limit
*Total coliforms*						
Nungwane	1,405.60	561.44/3,527.98	313.90	107.39/919.63	221.20	89.59/547.02
Senzakahle	1,405.60	561.44/3,527.98	278.10	117.46/659.73	27.80	11.74/65.95
Siyazenzela	22.10	8.96/54.68	22.10	8.96/54.68	22.10	8.96/54.68
Jabulani	943.50	349.79/2,551.18	140.60	56.12/352.65	22.10	8.96/54.68
*Fecal coliforms*						
Nungwane	313.90	107.39/919.63	2.60	1.13/6.20	1.40	0.55/3.47
Senzakahle	221.20	89.59/547.02	140	0.55/3.41	1.40	0.55/3.41
Siyazenzela	2.20	0.88/5.32	1.70	0.65/4.41	2.20	0.88/5.32
Jabulani	27.80	11.74/65.05	1.10	0.39/2.95	1.00	0.39/2.95
*E. coli*						
Nungwane	27.80	11.74/65.95	2.60	1.13/6.20	2.60	1.13/6.20
Senzakahle	22.10	8.96/54.68	2.60	1.13/6.20	1.40	0.55/3.47
Siyazenzela	1.40	0.55/3.47	n.d	-	n.d	-
Jabulani	2.60	1.13/6.20	1.10	0.39/2.95	n.d	-

* 95% confidence limits calculated according to Garthright and Blodgett [30] n.d—not detected (limit of detection is 0.2/g).

Though consensus has not been reached stipulating compost standards [11], the Code of Federal Regulations (CFR) from the US EPA proposes that biosolid based composts should have fecal coliforms levels not exceeding 1,000/g if it is to be used as fertilizer [33]. Similarly, the European Commission stipulates that composting products should not exceed 1,000 MPN/g for *E. coli* [34]. These standards were used to assess the bacterial numbers detected in the compost samples tested. All the compost samples from the four cooperatives met the American (<1,000 fecal coliforms/g) and European standard (<1,000 MPN/g *E. coli*). The respondents indicated that composting was an intricate process requiring a lot of time and patience. The making of compost involved the digging of shallow trenches that were filled by continuous layers of tree branches, green leaves and grass, wet animal manure, rotten food, cardboard, some watering and occasionally wood ash. Often, a hole is made in the middle using a pole and the height of the heap is at the discretion of the farmer. These compost heaps were abandoned for 3–6 months as according to the farmers it was virtually impossible to use the compost within this period as the compost was too hot and would therefore damage any seed planted. Discussions with key informants suggested that the making of a compost heap is a very physical process and as a result farmers may sometimes do it incorrectly. Incorrect composting may cause the maturing of the compost heap to be delayed as physico-chemical properties of the compost heap may not be conducive for the occurrence of the required biochemical reactions [35].

In October 2011 composts from all 4 cooperatives were 4 months old. However, the compost heaps were of different heights and were made up of varying amounts and combinations of manure. Farmers considered compost heap shrinkage and release of gas as indicators that it has cooled down and had reached maturity. According to Ryckeboer *et al*. [36], tools that measure levels of O_2_, moisture and temperature are necessary when determining composting stages and compost maturity. The uMbumbulu Agri-Hub cooperatives did not have these tools and therefore approximated compost maturity. However, fecal coliform levels met the US EPA standard set at <1,000/g for compost [33] as well as the *E. coli* levels specified by the European Commission [34]. The fecal coliform and *E. coli* levels decreased between October and December 2011, indicating that compost was not sufficiently mature and continued to mature over the 3 months analyzed, thereby leading to reduced levels of the hygiene indicators as a result of heat inactivation [36].

### 3.7. The Administration of Compost

Compost was administered into the soil in one of three ways: It was spread either on the top of the plot after the planting of seedlings, mixed with the soil prior to planting or added into individual holes before the planting of the seed. It was noted that there was some ambiguity about the science of composting amongst respondents. A number of farmers were aware of the decomposition aspect of composting but were unable to differentiate between materials that are readily decomposable and those that are not. Furthermore, farmers appeared to have a limited understanding of temperature variations within the compost heap. Several respondents admitted to depositing the feces of domestic animals on top of a maturing compost pile, this practice could lead to the transfer of pathogenic bacteria to the surface of the leafy vegetables during the addition of compost to the soil. According to Baldwin and Greenfield [35], the middle of the pile can reach temperatures of 63 °C which inactivates most non-endospore forming pathogens. Therefore, the feces of domestic animals (including pets like dogs and cats) added on top of the pile will not be sufficiently impacted by these high temperatures thereby increasing the risk of possible contamination. Respondents reported that composts containing manure from cattle attracted flies, pests and rodents including cockroaches and mice which led them to believe that compost may be a source of contamination as many flies and pests are vectors of disease. Respondents agreed that bacterial outbreaks would lead to customer loss as customers would lose trust and always question the hygienic quality of their vegetables.

### 3.8. The Use of Wet and Dried Manure (Umquba)

Besides its use in the making of compost, the respondents revealed that the use of fresh and dried manure was common amongst untrained farmers. The fresh manure was either used wet and mixed directly with the soil prior to planting seeds or the manure was firstly dried for approximately 3 weeks forming *Umquba* before being incorporated into the soil. A total of 24.7% of respondents admitted to using wet fresh manure directly on the soil while 50.7% of the respondents made *Umquba* (Table 2). The use of wet manure can be particularly dangerous as bovine feces have been identified as reservoir of pathogenic *E. coli* strains [37] which can cause foodborne diseases. Furthermore, the drying of manure may not be sufficient to completely eliminate pathogenic strains of *E. coli* such as STEC which are known to exhibit a low infectious dose [23,38].

### 3.9. Hygienic Quality of Leafy Vegetables

The MPN values for total and fecal coliforms fluctuated in the samples collected monthly from the four different locations in October, November and December 2011 but remained below 1/g for both lettuce and spinach. *E. coli* was not detected on the leaf surface (Table 6, Table 7).

**Table 6:** MPN per g of spinach for total and fecal coliforms as well as *E. coli* produced by different farmer cooperatives for the months of October, November, and December 2011.

Source of Spinach	October 2011		November 2011		December 2011	
	MPN/g	95% confidence interval lower/upper limit *	MPN/g	95% confidence interval lower/upper limit	MPN/g	95% confidence interval lower/upper limit
*Total coliforms*						
Nungwane	0.50	0.11/1.81	1.10	0.39/2.95	1.40	0.55/3.41
Senzakahle	1.40	0.55/3.47	1.40	0.55/3.47	1.10	0.39/2.48
Siyazenzela	0.70	0.22/2.15	0.70	0.22/2.15	0.80	0.24/2.95
Jabulani	0.20	0.028/1.41	1.40	0.55/3.47	1.40	0.55/3.47
*Fecal coliforms*						
Nungwane	0.20	0.028/1.41	0.70	0.22/2.15	0.50	0.11/1.81
Senzakahle	0.70	0.22/2.15	0.70	0.22/2.15	0.50	0.11/1.81
Siyazenzela	0.20	0.028/1.41	0.20	0.028/1.41	n.d	-
Jabulani	n.d	-	0.40	0.11/1.81	n.d	-
*E. coli*						
Nungwane	n.d	-	n.d	-	n.d	-
Senzakahle	n.d	-	n.d	-	n.d	-
Siyazenzela	n.d	-	n.d	-	n.d	-
Jabulani	n.d	-	n.d	-	n.d	-

* 95% confidence limits calculated according to Garthright and Blodgett [30] n.d—not detected (limit of detection is 0.2/g).

**Table 7:** MPN per g of lettuce for total and fecal coliforms as well as *E. coli* produced by different farmer cooperatives for the months of October, November, and December 2011.

Source of Lettuce	October 2011		November 2011		December 2011	
	MPN/g	95% confidence interval lower/upper limit *	MPN/g	95% confidence interval lower/upper limit	MPN/g	95% confidence interval lower/upper limit
*Total coliforms*						
Nungwane	0.50	0.11/1.81	0.20	0.028/1.41	0.50	0.11/1.81
Senzakahle	1.40	0.55/3.47	0.70	0.22/2.15	n.d	-
Siyazenzela	1.40	0.55/3.47	0.90	0.34/2.50	0.70	0.22/2.15
Jabulani	0.70	0.22/2.15	0.70	0.22/2.15	0.70	0.22/2.15
*Fecal coliforms*						
Nungwane	n.d	-	n.d	-	0.20	0.281.41
Senzakahle	0.40	0.11/1.62	0.2	0.028/1.41	n.d	-
Siyazenzela	0.70	0.22/2.15	0.68	0.22/2.15	0.20	0.028/1.41
Jabulani	n.d	-	0.20	0.028/1.41	0.40	0.10/1.62
*E. coli*						
Nungwane	n.d	-	n.d	-	n.d	-
Senzakahle	n.d	-	n.d	-	n.d	-
Siyazenzela	n.d	-	n.d	-	n.d	-
Jabulani	n.d	-	n.d	-	n.d	-

* 95% confidence limits calculated according to Garthright and Blodgett [30] n.d—not detected (limit of detection is 0.2/g).

Though microbiological quality of raw fruit and vegetables is not sufficiently covered by current South African legislation, the DOH [39] recommends that raw fruits and vegetables should have total coliform levels not exceeding 200/g. The lettuce and spinach sampled (Table 6 , Table 7) had total coliform levels <2/g at the time of sampling, thereby meeting this requirement. In addition, no presumptive *E. coli* was detected. For organic lettuce produced in Spain it was reported that more than 75% of produce samples analyzed exhibited MPN values for presumptive *E. coli* of <30/100 g [10]. Further to this, all vegetables produced by the farmers are thoroughly rinsed with uMbumbulu municipal tap water at the Agri-Hub which will meet the strict (no detectable fecal coliforms per 100 mL) drinking water standards established for South Africa by the Department of Water Affairs and Forestry [40]. The additional rinsing with uncontaminated tap water can improve the microbiological quality of the produce [41,42]. Although this rinsing is therefore a useful procedure, it is important that measures that prevent contamination of produce are employed at all stages of production as pathogen internalization may occur rendering rinsing with water less effective [18].

According to the Department of Water affairs and Forestry, water quality is considered as a determinant of the microbial quality of the final vegetable product [32]. The use of uncontaminated irrigation water sources such as tap water is indeed important as potential links between water quality and microbiological quality of produce have been suspected [15]. In addition, as long as drip irrigation is not available, farmers should be advised to irrigate produce close to the root area, as surface irrigation increases chances of contamination especially if irrigated with water of unknown microbiological quality [3,7]. Additional measures such as improving the composting process by applying a physical cover to increase the temperature [12] and improved pre- and post-harvest hygiene practices [43] can be expected to further improve the quality of the final product.

## 4. Conclusions

Based on the data for the selected bacterial hygiene indicators, the uMbumbulu Agri-Hub produces vegetables that at the time of analysis were meeting the total coliform levels (<200/g) stipulated as acceptable by the DOH for raw fruits and vegetables. Microbiological analysis of irrigation water showed that the MPN values were meeting South African sand WHO standards for safe irrigation. All four cooperatives met the US EPA and European Commission compost standard for fecal coliforms and *E. coli*. However, the need for farmers to gain access to tools that can assist them in determining compost maturity was highlighted. This is important given that manure or compost not meeting these standards might be added as fertilizer to soil. In this case consumers might be put at risk due to the transfer of potentially pathogenic bacteria from soil onto minimally processed vegetables. As there are limited numbers of training courses that focus specifically on microbiological quality in the farming environment, farmers would benefit from training on good hygiene practices throughout the farming supply chain.

## Supplementary Files

Supplementary File 1 Supplementary (PDF, 190 KB)
