# Supplementary material for: Appraisal of Hygiene Indicators and Farming Practices in the Production of Leafy Vegetables by Organic Small-Scale Farmers in uMbumbulu (Rural KwaZulu-Natal, South Africa)
Source: Int J Environ Res Public Health. 2013 Sep 13;10(9):4323–38. doi: 10.3390/ijerph10094323 (PMC3799505; doi:10.3390/ijerph10094323)
Supplement: Supplementary File 1 — Supplementary (PDF, 190 KB) [file ijerph-10-04323-s001.pdf]

Investigation of Selected Hygiene Parameters of uMbumbulu Small-scale Farmers' Organic Produce (leafy salad vegetables) and Subsequent Identification of Factors Affecting Farmer Practices and Food Security

Questionnaire

Section 1- Demographics

1.1 Ubulili

Ubulili	X
Umuntu Wesilisa	
Umuntu wesfazane	

1.2 Iminyaka

Iminyaka	X
a. 20<	
b. 21-39	
c. >40	

1.3 Izinga lemfundo

Izinga	X
a. Grade 7<	
b. Grade 8- Grade 12	
c. >Grade 12	
d. Angikaze ngiye eskoleni	

1.4 Niyitholaphi imali

Indawo	Yebo	Cha
a. Remittance		
b. Ukulima<br>c. Social Grant		
d. iMpesheni		
e. Uyasebenza		
f. Okunye		

1.5 Ingakanani imali uyenzayo ngokulima ngeviki uma uvunile?

Imali (ZAR)	X
a. 0-250	
b. 251-500	
c. 501-1000	
d. >1001	

Section 2- Sub problem 1

Which vegetables are produced?

2.1 Kungani ukhethe ukulima ngendlela e organic? Khetha konke ovumelana nakho

Isizatho	Yebo	Cha
a. Abathengi bathanda lenhlobo yokulima		
b. Iyonga imali		
c. Iphatha imvelo kahle		
d. Ukuthi umndeni udle		
e. Okunye		

2.2 Iziohi izithelo ozitshalayo?

Isithelo	Yebo	Cha	Isithelo	Yebo	Cha
a. Carrot			f. Beetroot		
b. Green beans			g. Onion		
c. Potato			h. Lettuce		
d. Spinach			i. Cabbage		
e. Tomato			j. Swiss Chard		

Kungani utshala lezizithelo?

____

____

____

2.3 Uzidayisela Obani?

____

____

____

2.4 Uyazidla lezizithelo ekhaya lakho? Y / C

2.5 Emva kokuzidla ekhaya, engabe uhleze uba nezithelo ezisalayo ukuze udayise? Y / C

<u>Section 3- Sub problem 2</u>

What is the safety of the salad vegetables?

3.1 Engabe uyabusebenzisa ubulongwe bezilwane uma ulima? Y / C

3.2 Ubulongwe baziphi izilwane obusetshenziswayo? Tick all that apply

	Animals	Yebo	Cha
a.	Izinkukhu		
b.	Izimbuzi		
c.	Izinkomo		
d.	Okunye		

3.3 Engabe ubuthathaphi lobubulongwe?

____

____

3.4 Kungani kusetshenziswa lobubulongwe?

____

____

____

3.5 Engabe yini oyenzayo kulobubulongwe ukuze kugcine buyi compost? Chaza kabanzi

____

____

3.6 Le compost isetshenziswa kanjani ensimini? Chaza kabanzi

____

____

____

3.7 Inani lecompost osetshenziswayo likalwa yini? Y / C

Uma uvumelani, kanjani?

____

____

3.8 Aqhamukaphi amanzi okuchela?

**Source**	**Yebo**	**Cha**
a. Amanzi kampompi		
b. Amanzi ethanki		
c. umfula/borehole/stream/wetland (natural water sources)		
d. Other (isphethu)		

3.9 Engabe lawamanzi ayahlanzwa ngendlela ethize? Khetha okukodwa

**Process**	**X**
a. Yebo, ayahlanzwa	
b. Cha, kuchelelwa ngawo engahlanziwe	
c. Okunye	

3.10 Uma uphendule ngo (a) ahlanzwa kanjani?

____

____

____

3.11 Ikuphi okwenzayo phakathi kwalokhu okulandelayo ukuqiniseka ukuthi uhlanzekile? Khetha konke ovumelana nakho

Process	Yebo	Cha
a. Ngigeza ama bhuzu/ amadadla		
b. Ngiwasha onke ama thuluzi		
c. Angenzi lutho, ngiya ensimini nginje		
d. Okunye		

3.12 Uma uvuna, kukhona izitshalo ezihuzukayo noma ezilimalayo? Y / C

3.13 Uzenzenjani izitshalo ezihuzukile noma ezilimele?

Procedure	Yebo	Cha
a. Uzidayisela omakhelwane		
b. Ziyadayiswa, kodwa ngenani eliphansi		
c. Ziyalahlwa		
d. Zisetshenziswa ekhaya		
e. Okunye		

3.14 Engabe izitshalo zakho ziyahashazwa emanzini ngaphambi kokupakishwa? Y / C

Uma kunjalo, engabe zihashazwa ngamanzi ochelelwa ngawo? Y / C

Section 4- Sub Problem 3

Which areas require capacity building in microbial safety?

4.1 Usuke wafundiswa ngokutshalwa organically? Y / C

Uma kunjalo khetha itraining programme. Khetha konke ovumelana nakho

Programme	Yebo	Cha
a. Composting (ukwenza umquba)		
b. Post-harvest protocol (ukuphatwa kwezitshalo emva kokuvuna)		
c. Water safety (okuhlanzeka kwamanzi)<br>d. Types of sustainable agriculture (izinhlobo zolimi lwenvelo)		
e. Good personal hygiene practices (ukuhlanzeka komuntu)		
f. Pest control (ukubulala izinambuzane)		
g. Soil management (ukunakekelwa komhlabathi)		
h. Soil preparation (ukulungisa umhlabathi)		
i. Okunye		

4.2 Uba owaye fundisa?

Trainer	Yebo	Cha
a. Government		
b. Private company		
c. NGO		
d. Okunye		

4.3 Ngolwakho ulwazi, ikuphi phakathi kwalokhu okulandelayo okungaba isisusa samagcikwane?

Tick all that apply

Source	Yebo	Cha
a. Water		
b. Soil		
c. Tools(amathuluzi)		
d. Compost		
e. Other		
f. Don't know		

4.4 Iziphi izizathu ezenza lokhu okukhethile isisusa samagcikwane?

____

____

____

4.5 Ngowakho umbono ikuphi okulandelayo okungadala ukuthi izitshalo zigcine zinamagcikwane?

Khetha konke ovumelana nakho

Practice	Yebo	Cha
a. Ukusetshenziswa kwamanzi anamagcikwane		
b. Icompost engenziwanga kahle		
c. Ukungahlanzekio yomuntu		
d. Umhlabathi onamagcikwane		
e. Amathuluzi anamagcikwane		
f. Don't know		

4.6 Ibuphi ubungozi obungadaleka uma izitshalo ezinamagcikwane zithengiswa? Khetha konke ovumelana nakho

Potential Hazard	Yebo	Cha
a. Ukuguliswa amagcikwane		
b. Abukho ubungozi		
c. Angeke uthembeke kumakhasimende		
d. Other		
e. Don't know		

4.7 Zikhona izifo ozaziyo ezihlangene nezitshalo ezikezahlasela umphakathi emphakathini? Y / N

4.8 Ngokwakho ukwazi engabe kukhona ukuqwashiswa ngokwanele ngokuphepha kwezitshalo emphakathini? Y / N

Uma kungenjalo yini engenziwa?

____

____

____

4.9 Iyiphi itraining ocabanga ukuthi uyayidinga ephathelene namagcikwane ezitshalweni? Chaza kabanzi

____

____

____

Investigation of Selected Hygiene Parameters of uMbumbulu Small-scale Farmers' Organic Produce (leafy salad vegetables) and Subsequent Identification of Factors Affecting Farmer Practices and Food Security

Questionnaire

Section 1- Demographics

1.1 Gender

Gender	X
Male	
Female	

1.2 Age

Age	X
a. 20<	
b. 21-39	
c. >40	

1.3 Level of education

Level	X
a. Grade 7<	
b. Grade 8- Grade 12	
c. >Grade 12	
d. No formal education	

1.4 Sources of income: Tick all that apply

Source	Yes	No
a. Remittance		
b. Farming		
c. Social Grant		
d. Pension		
e. Salaried Job/Wages		
f. Other		

1.5 How much do you make from organic farming weekly during harvest time?

Amount (ZAR)	X
a. 0-250	
b. 251-500	
c. 501-1000	
d. >1001	

<u>**Section 2- Sub problem 1**</u>

Which vegetables are produced?

2.1 Why do you practice organic farming? Tick all that apply

Reason	Yes	No
a. Market demand of organic produce		
b. Cost effective		
c. Its gentle on the environment		
d. To ensure food security		
e. Other		

2.2 Which vegetables do you plant?

Vegetable	Yes	No	Vegetable	Yes	No
a. Carrot			f. Beetroot		
b. Green beans			g. Onion		
c. Potato			h. Lettuce		
d. Spinach			i. Cabbage		
e. Tomato			j. Swiss Chard		

Why do you plant these vegetables?

____

____

____

2.3 Who do you mainly supply?

____

____

____

2.4 Do you use the products yourself? Y / N

2.5 Do you always have a surplus to sell? Y / N

Section 3- Sub problem 2

What is the safety of the salad vegetables?

3.1 Do you use animal manure in your farming activities? Y / N

3.2 Which type of waste is used? Tick all that apply

	Animals	Yes	No
a.	Chicken		
b.	Sheep		
c.	Cow		
d.	Other		

3.3 Where do you get it from?

____

____

3.4 Why is this type of waste used?

____

____

____

3.5 How do you prepare the animal manure? Please explain.

____

____

____

____

____

____

3.6 How is this manure/compost used in the plot? Please explain

____

____

____

3.7 Is the quantity of the manure/ compost used measured in any way? Y / N

If yes, how?

____

____

3.8 What is the source of irrigation water?

Source	Yes	No
a. Tap water		
b. Tank water		
c. River/borehole/stream/wetland (natural water sources)		
d. Other		

3.9 Is the water used for irrigation treated? Please tick most relevant answer

Process	X
a. Yes, it is treated before watering plants	
b. No, it is applied directly to plants without treatment	
c. Other	

3.10 In the case of (a) how is this water treated?

____

____

____

3.11 Do you adhere to any of the following with regards to personal hygiene before going to the garden? Tick all that apply

Process	Yes	No
a. Wash your hands and gum boots		
b. Wash all equipment required		
c. Go to the garden as you are		
d. Other		

3.12 When harvesting, are there vegetables which are bruised or damaged? Y / N

3.13 What do you do with the bruised or damaged vegetables?

Procedure	Yes	No
a. Sold to neighbours		
b. Sold to market but at a discount price		
c. Discarded		
d. Taken for personal use		
e. Other		

3.14 Are vegetables rinsed before packaging for the market? Y / N

If yes, are they rinsed with the same water used for irrigation? Y / N

Section 4- Sub Problem 3

Which areas require capacity building in microbial safety?

4.1 Have you received any form of training in organic farming? Y / N

If yes, please name training program. Tick all that apply

Programme	Yes	No
a. Composting		
b. Post-harvest protocol		
c. Water safety<br>d. Types of sustainable agriculture		
e. Good personal hygiene practices		
f. Pest control		
g. Soil management		
h. Soil preparation		
i. Other		

4.2 Who provided the training?

Trainer	Yes	No
a. Government		
b. Private company		
c. NGO		
d. Other		

4.3 According to your knowledge, which of the following can be a source of bacterial contamination? Tick all that apply

Source	Yes	No
a. Water		
b. Soil		
c. Tools		
d. Compost		
e. Other		
f. Don't know		

4.4 Why are these possible sources of contamination?

____

____

____

4.5 In your opinion, which of the following practices can compromise the microbial safety of produce? Tick all that apply

Practice	Yes	No
a. Use of contaminated water		
b. Poor composting techniques		
c. Poor personal hygiene		
d. Contaminated soils		
e. Contaminated equipment		
f. Don't know		
g. Other		

4.6 What potential hazards may this have on the market? Tick all that apply

Potential Hazard	Yes	No
a. Bacterial outbreaks		
b. No hazards		
c. Lose trust of customers		
d. Other		
e. Don't know		

4.7 Do you have any knowledge of outbreaks/sickness linked to harvested fresh produce in your community? Y / N

4.8 According to your knowledge, is there enough awareness about produce safety in your community? Y / N

If no, what do you think should be done to improve awareness?

____

____

____

4.9 What training related to safety of vegetables do you think you need? Please explain

____

____

____
